# Cost-effective DNA methylation profiling by FML-seq

**DOI:** 10.1101/2023.01.13.523849

**Published:** 2023-01-14

**Authors:** Joseph W. Foley, Shirley X. Zhu, Robert B. West

**Affiliations:** 1 Department of Pathology, Stanford University School of Medicine, Stanford, California, USA

## Abstract

Current methods for profiling DNA methylation require costly reagents, sequencing, or labor time. We introduce FML-seq, a sequencing library protocol that greatly reduces all these costs. Relative to other techniques tested on the same human cell lines, FML-seq produces similar measurements of absolute and differential cytosine methylation at a fraction of the price. FML-seq enables inexpensive, high-throughput experimental designs for large-scale epigenetics research projects.

## Introduction

DNA methylation is a crucial and well-studied epigenetic mark in many eukaryotes [1]. However, DNA methylation profiling remains one of the only areas of genomics where sequencing has not fully displaced microarrays. Each sequencing-based technique has its own impracticalities. Like the microarrays, several common approaches use deamination to distinguish cytosine from methylated cytosine. Whole-genome bisulfite sequencing (WGBS) [2] simply deaminates the entire genome, and deep whole-genome sequencing is then required to count modified vs. unmodified bases at every cytosine. Reduced-representation bisulfite sequencing (RRBS) [3] reduces the sequencing cost by first using a methylation-insensitive restriction endonuclease to enrich for genome fragments with high CpG content, but this requires a more cumbersome protocol for library preparation. Other approaches such as methylated DNA immunoprecipitation (MeDIP-seq) [4] directly enrich methylated cytosine and forgo deamination, but this depends on the specificity of each batch of antibody. Here we introduce FML-seq, a method that fills the gap for rapid, high-throughput, and cost-effective DNA methylation profiling.

## Results

### The FML-seq method

**Figure 1:**
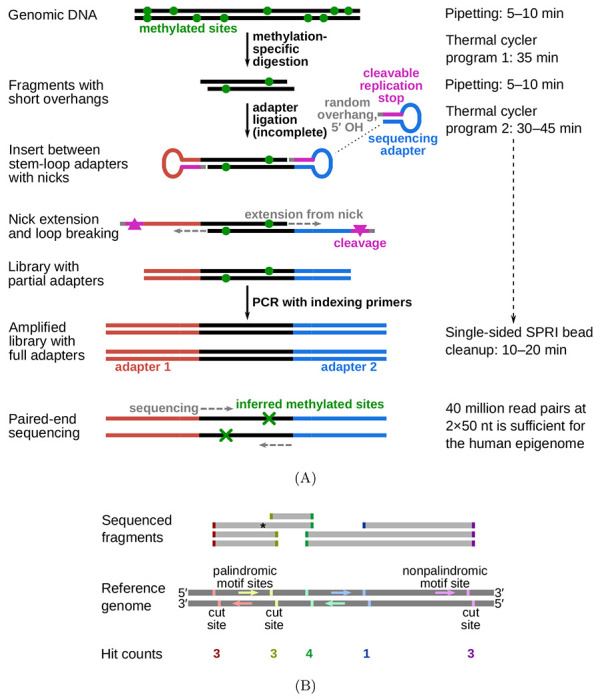
Diagram of FML-seq. A: Library preparation reactions. Genomic DNA is digested by a methylation-dependent restriction endonuclease that cuts at a known distance from the methylated cytosine in its motif and leaves a short single-strand overhang of unknown bases. Stem-loop (hairpin) sequencing adapters with complementary random overhangs are ligated to the digested gDNA fragments, but the phosphodiester backbone is completed only on one strand because the adapters lack a 5′ phosphate. The resulting single-strand nick is extended by DNA polymerase to fill in a second strand complementary to the adapter’s loop, while the unneeded stem strand is degraded. This library of gDNA inserts between double-stranded linear short adapters is then amplified by standard polymerase chain reaction with long indexing primers to produce a sequencing-ready library. A standard solid-phase reversible immobilization (SPRI) bead cleanup without size selection is sufficient to purify the library. Paired-end sequencing reads imply the location of the two methylated cytosines resulting in each observed fragment. B: Counting FML-seq fragments as hits at methylated motif sites. The restriction endonuclease used here, MspJI, cuts at the motif ^m^CNNR. Each copy of this motif on either strand implies a potential cut site at a certain distance past its 3′ end. When paired-end sequence reads are aligned to the reference genome, each end of a sequenced fragment counts as one hit for the corresponding motif site; for example, the fragment marked by an asterisk tallies one hit each for the red and green motif sites. The number of hits for a given motif site corresponds to the fraction of genome copies methylated at that motif’s cytosine position.

The protocol for fragmentation at methylated loci and sequencing (FML-seq) comprises only three steps (Figure 1A). First, genomic DNA (gDNA) is digested by a methylation-dependent restriction endonuclease that cuts at a certain distance from the 5-methylcytosine or 5-hydroxymethylcytosine in its motif and leaves a short overhang [5]. Second, a master mix is added with combined reagents for sticky-end adapter ligation, preparation of the specially designed adapters [6], and indexing PCR. Finally, a single cleanup without size selection is sufficient to purify the library, because the digestion does not produce unusably short fragments (Figure S1) and the adapter design prevents byproducts without gDNA inserts (see Supplemental Text, Figure S2). The resulting library contains unaltered genome sequences alignable by standard pipelines. Each end of a library fragment is derived from a methylation-dependent digestion, so paired-end sequencing detects two methylated cytosine positions per fragment (Figure 1B).

Unlike the deamination methods, which produce dual-channel data by counting converted and unconverted bases at each position, FML-seq produces single-channel data, in which the total number of reads aligned at a given genome position corresponds to the amount of methylated cytosine present in the sample. Previous techniques have also used methylation-dependent digestion [5] but FML-seq represents a simplification of those protocols (see Supplemental Text).

### Specificity of FML-seq

FML-seq’s specificity for methylated cytosine depends on the endonuclease. Concordant with previous reports [5], digestion of unmethylated gDNA from lambda bacteriophage yielded no detectable library product, unless greatly overamplified (Figure S3A). Reads from methylated gDNA aligned largely at the sequence motif targeted by Dcm methylase [7] as expected; reads from unmethylated gDNA had little enrichment for the methylase’s motif but still aligned at the endonuclease’s motif [5] (Figure S3B).

The other factor in FML-seq’s specificity is whether other kinds of gDNA fragmentation produce ligatable ends. To fit the adapters, a fragment must have a 5′ overhang of 4 nt with a terminal phosphate, which is produced by the endonuclease but unlikely to result from other kinds of fragmentation. Accordingly, no-endonuclease control libraries using gDNA from either fresh cells or degraded archival tissue showed no detectable library product unless greatly overamplified (Figure S4).

### Biological validation

To compare FML-seq with other methods, we prepared libraries from four well-studied human cell lines (Figure S5). The sequence reads from human gDNA had high alignability to the reference genome (Figure S6A). Insert lengths varied widely beyond the canonical 32 bp semipalindromic fragment (Figure S1C) [5], but very few inserts were too short to align (Figure S7). Most reads aligned at the expected sequence motif (Figure S6B) [5].

**Figure 2:**
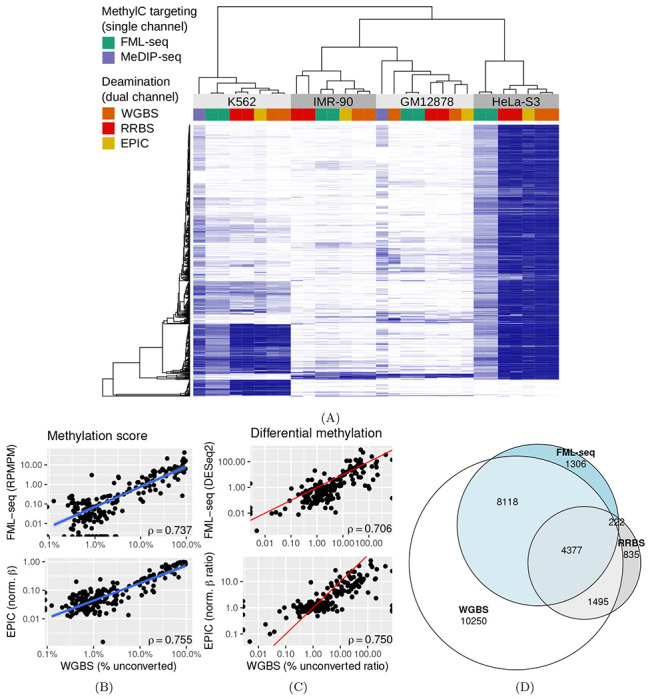
FML-seq recovers a similar biological signal as more costly DNA methylation profiling methods on the same samples. A: Normalized methylation signal (blue: EPIC *β*, WGBS and RRBS percent methylation, MeDIP VSD, FML-seq log RPMPM) at the 500 promoters with the most variation among cell lines according to WGBS. Technical replicates are shown separately. B: Methylation scores by promoter for K562 in FML-seq or EPIC vs. WGBS, replicates pooled. Only 200 randomly sampled promoters are graphed and used for the blue least-squares regression line but Spearman’s *ρ* is calculated with all promoters. C: Methylation fold change by promoter for K562 vs. HeLa-S3 in FML-seq or EPIC vs. WGBS, replicates pooled. The same subset of promoters are graphed as in B. The red line shows *y* = *x*. D: Overlap of significantly (BH-adjusted *p* < 0.01) differentially methylated promoters between K562 and HeLa-S3 according to WGBS (24,240 significant), RRBS (6,929), and FML-seq (14,023).

Within gene promoters, FML-seq signal correlated well with DNA methylation signals detected by other methods (Figure 2A): the two-channel deamination methods (EPIC, WGBS, RRBS) were most similar to one another but FML-seq was more similar to them than another one-channel method (MeDIP-seq). FML-seq showed lower methylation than other methods for HeLa-S3 in particular, as widespread hypermethylation diluted the one-channel signal, though such extreme genome dysregulation may not be observed in many experiments. FML-seq’s absolute signal per promoter (Figure 2B) and estimated fold change (Figure 2C) were correlated with the most comprehensive method, WGBS to a similar degree as the EPIC microarray was correlated with WGBS. Absolute signal may be confounded by promoter-to-promoter variation in the density of restriction motif sites, but the typical goal of DNA methylation profiling is detecting relative signal, sample-to-sample differential methylation, at each individual locus. The list of differentially methylated promoters detected by FML-seq showed concordance with WGBS and greater sensitivity than RRBS (Figure 2D).

### Minimal sequencing conditions

Serial dilutions from 60 ng gDNA input showed successful but lower-quality results down to 6 ng (Figure S10). Because FML-seq analysis counts entire sequence reads rather than bases within each read, long reads are no more useful than short reads as long as they can be confidently aligned to the reference genome; the shortest available read lengths proved sufficient (Figure S9). Given the diminishing returns of additional sequencing depth (Figure S10), sufficient sequencing for a human gDNA sample is roughly 40 million read pairs, or 96 libraries per NovaSeq S2 flow cell. Thus although sequencing is the main cost of the FML-seq workflow at current prices, that expense is also more economical than other approaches.

## Discussion

By profiling genome-wide DNA methylation more cost-effectively than current methods, FML-seq will allow new studies with a larger scale of samples than financially feasible with previous methods, while the short protocol in 96-well plates will significantly reduce labor and risks of error (Table 1). Recently new reductions in sequencing prices have been forecast to bring the “$100 genome” [8]; as sequencing makes up nearly all the cost of FML-seq, cheaper sequencing could also bring the $20 epigenome. Whereas somatic genome sequencing might be informative only once per individual, inexpensive epigenome profiling by FML-seq could be used repeatedly to track diverse variables such as disease progression, tissue differentiation, or genomic response to the environment. With FML-seq, epigenetic profiling could become a routine high-throughput assay like qPCR rather than a costly exploratory experiment limited to precious samples.

## Materials and methods

### Data generation

#### Reference genomic DNA preparation

HeLa-S3, IMR-90, and K562 gDNAs were purchased from MilliporeSigma (catalog #87110901, 85020204, 89121407). GM12878 gDNA was purchased from the Coriell Institute for Medical Research (#NA12878). Methylated lambda bacteriophage gDNA, from strain cI857 Sam7 grown in *E. coli* strain W3110, was purchased from Thermo Fisher Scientific (#SD0011). Unmethylated lambda gDNA, from the same virus strain grown in *dam*^−^ *dcm*^−^ *E. coli* strain GM2163, was purchased from Thermo Fisher Scientific (#SD0021). All gDNA samples were requantified with a Qubit 1X dsDNA High Sensitivity kit (Thermo Fisher Scientific #Q33230) and diluted according to this measurement in nuclease-free 1X TE buffer with 0.05% w/v Tween 20. All experiments used 60 ng gDNA except those comparing lower inputs, for which gDNA was serially diluted and technical replicates were taken from the same dilution.

#### FML-seq library preparation

Sequencing libraries were prepared according to the FML-seq protocol (Supplemental Protocol) with final holds at 14°C. In the specificity validation experiments, technical replicates were performed side-by-side with different numbers of PCR cycles in order to detect widely different yields; in these and the experiments comparing different amounts of gDNA, at the end of each measured cycle number before the last, the appropriate tubes were transferred to a dry-block incubator at 72 °C for 1 min and then to a 4 °C refrigerator to replace the final extension step in the PCR program. In the no-endonuclease control experiment, MspJI restriction endonuclease was replaced by an equal volume of its storage buffer (New England Biolabs #B8002S).

#### Library quality control and quantification

Libraries from all experiments were profiled, undiluted, on an Agilent TapeStation 4200 with the High Sensitivity D1000 Reagents (#5067-5585). Libraries to be sequenced were diluted to 1/10,000 concentration by serial 1/100 dilutions and quantified by qPCR with the primers 5′-AATGATACGGCGACCACCGA-3′ and 5′-CAAGCAGAAGACGGCATACGA-3′ at 450nM each, using PowerUp SYBR Green Master Mix (Thermo Fisher Scientific #A25742) and a program of 2 min at 50 °C, 2 min at 95 °C, then 40 cycles of 15 s at 95 °C, 1 min at 60 °C. The KAPA Library Quantification DNA Standards #1–5 (Roche #07960387001) were used for the standard curve. The qPCR measurements were scaled by the qPCR’s dilution factor and the TapeStation’s average library molecule lengths to calculate the library molarities used for pooling.

#### Sequencing

Shallow sequencing with long reads, for initial characterization of the libraries from the methylated vs. unmethylated lambda gDNA experiment and the human cell-line gDNA experiment, was performed on an Illumina MiSeq with the MiSeq Reagent Nano v2 kit (#MS-103-1001) yielding paired-end reads of 2×154 nt and index reads of 2×8 nt, and all libraries from each experiment loaded at equal molarity. Deeper sequencing with short reads was performed for the 60 ng libraries from the human cell-line experiment; these were deliberately sequenced to unnecessary depth in order to enable rarefaction analysis (see Supplemental Protocol for sequencing recommendations) using an Illumina NovaSeq 6000 with the S1 Reagent Kit v1.5 (#20028319) yielding paired-end reads of 2×61 nt and index reads of 2×8 nt. The 6 ng and 600 pg libraries were sequenced on an Illumina NextSeq 500 with the High Output Kit v2.5 (#20024906) yielding paired-end reads of 2×38 nt and index reads of 2×8 nt.

## Data processing

### Promoter annotations

The comperehensive set of 40,891 non-overlapping “promoter-like” candidate cis-regulatory elements (cCREs) in the human genome [9] was converted from the hg38 reference genome to T2T-CHM13v2.0 (hs1) [10] by UCSC liftOver [11], eliminating cCREs whose old coordinates were deleted in the new reference. Only cCREs with lengths of 150 to 350 bp in the new reference were kept, matching the range in the old reference. These filters yielded a final set of 40,351 promoters. Each sequence motif or microarray probe was counted within a cCRE if the position of the cytosine base being tested for methylation state was within the boundaries of the cCRE, even if the rest of the motif or probe sequence lay beyond the boundaries.

### FML-seq

Standard Nextera adapter sequences were trimmed from all FML-seq reads by cutadapt version 4.1 [12] and the NextSeq trimming option was enabled for NextSeq data. Trimmed reads from human samples were aligned to the T2T-CHM13v2.0 (hs1) reference genome [10], and from lambda bacteriophage samples to the circularized lambda reference genome (RefSeq NC_001416), by bwa-mem2 version 2.2.1 [13]. An FML-seq read was counted as a hit at a CGNR site if the beginning of the read (the end of the fragment) aligned at the expected distance of 10 bp from the motif (to the right of a motif on the forward strand or to the left on the reverse strand). The hit count for each promoter was calculated as the total of all CGNR positions whose first C base was within the promoter. For exploratory analysis, each promoter’s hit count was normalized both by the library size and by the number of CGNR motifs within the promoter to reads per million per motif (RPMPM): for any promoter *i* with hit count *c_i_* and motif count *m_i_*,

$${\text{RPMPM}}_{i}=\frac{10_{c_{i}}^{6}}{m_{i}\sum_{j}c_{j}},$$

and this value was added to 1 before computing the logarithm, log_10_(RPMPM_*i*_ + 1).

### MeDIP-seq

Standard TruSeq adapter sequences were trimmed from all FML-seq reads by cutadapt version 4.1 [12], then the trimmed reads from human samples were aligned to the T2T-CHM13v2.0 (hs1) reference genome [10] by bwa-mem2 version 2.2.1 [13]. Each DNA fragment inferred from the alignments of paired-end reads was counted as a hit in a given promoter if the center position of the fragment was within the boundaries of the promoter. The matrix of promoters × samples was processed in DESeq2 version 1.36 [14] with no design variable, as the experiments were unreplicated, and normalized with the variance-stabilizing transformation.

### Bisulfite sequencing

Reads from WGBS and RRBS were processed with similar pipelines. An extraneous sequencing run that was previously concatenated into one of the FASTQ files, ENCODE accession number ENCFF000MHC, was removed from that file as it lacked the characteristic base composition of bisulfite conversion. Standard TruSeq adapter sequences were trimmed from all reads by cutadapt version 4.1 [12], then the trimmed reads were aligned to the converted T2T-CHM13v2.0 (hs1) reference genome [10] by bwa-meth version 0.2.5 using bwa-mem2 version 2.2.1 [13]. For RRBS reads, the two bases before each adapter were also trimmed as these are not subject to bisulfite conversion, and the minimum score was set to zero in bwa-mem2 because of the short reads (36 nt). Converted and unconverted bases were counted at each CpG position by MethylDackel version 0.6.1 and the methylation score of each promoter was calculated as the total proportion of methylated bases detected at all CpG positions within the promoter, equivalent to the mean proportion of methylation across positions weighted by the coverage at each position.

### Microarray

Genome coordinates of CpG sites targeted by microarray probes were converted from the hg38 reference genome to T2T-CHM13v2.0 (hs1) [10] by UCSC liftOver [11], eliminating sites whose old coordinates were deleted in the new reference or whose base sequence was no longer CG in the new reference. The previous samples from ENCODE were imported in one batch by ChAMP version 2.26.0 [15]. The methylation score of each promoter was calculated as the mean beta value of all probes measuring a cytosine position within the promoter.

### Method clustering

A subset of 9,876 promoters were measurable by every method (at least one EPIC probe, WGBS/RRBS CpG read, MeDIP fragment, or 60 ng FML-seq CGNR read). From this subset, the 500 promoters with the greatest variation among cell lines were selected by the greatest *χ*^2^ scores from the two-way table of cell type × methylated vs. unmethylated hit counts from WGBS with replicates pooled. Each method’s 500 promoters × samples matrix of methylation scores (EPIC *β*, WGBS and RRBS percent methylation, MeDIP VSD, FML-seq log RPMPM) was linearly scaled to the range [0, 1] before the matrices were concatenated. Hierarchical clustering was perfomed by UPGMA on Pearson distances (1 − *r*) of the concatenated matrix.

### Data access

The sequencing data generated in this study are available in the NCBI BioProject database under accession number PRJNA914781. Data processing pipeline scripts are collected at https://github.com/jwfoley/FMLtools. Scripts used to perform the analyses in this study are collected in Supplemental Code.

## Supplementary Material

Supplement 1

Supplement 2

Supplement 3

Supplement 4

## Figures and Tables

**Table 1:** FML-seq requires less labor and reagent cost than representative cytosine-methylation profiling methods. Cost per sample includes all reagents but not standard consumables (tubes, pipet tips) and is rounded to the nearest 5 USD. Protocol time includes all steps from isolated gDNA to sequencing-ready library. “Special equipment” for sample preparation excludes common instruments such as pipets, thermal cyclers, centrifuges, and separation magnets. Sequencing read lengths are per the manufacturers’ recommendations. Deamination-based sequencing methods use 30X coverage per ENCODE guidelines, assuming 80% read alignment to the human genome. Sequencing costs use list prices for appropriate Illumina NovaSeq 6000 High Output reagents with up to 96-plex indexing. CpG sites are counted in the CHM13v2 reference sequence, both strands.

	Microarray (Illumina)	WGBS (IDT)	EM-seq (NEB)	RRBS (Zymo)	Targeted BS (Illumina)	FML-seq
Reagent cost	$265	$90	$40	$45	$280	$5
Protocol time	4 days	1 day	2 days	2 days	2 days	2 hours
Limiting scale	8-sample chip	24-tube centrifuge	96-well plate	24-tube centrifuge	24-tube centrifuge	96-well plate
Special equipment	Incubators and water circulator	Sonicator	Sonicator		Sonicator	
Minimum gDNA	250 ng	100 pg	10 ng	10 ng	500 ng	6 ng (1000 cells)
Sequencing reads		2×150 nt ×375M	2×150 nt ×375M	2×50 nt ×90M	2×100 nt ×55M	2×50 nt ×40M
Sequencing cost		$615	$615	$180	$135	$80
Targeted CpG sites	0.85M	68M	68M	5M	3M	36M
